# Albugo

**Published:** 1854-12

**Authors:** 


Albugo. Electro-puncture.—Dr. D. Tavignot (*Bull. de Thér*, *Juillet*, p. 49,) relates the following: A young girl, aged 19, was attacked with catarrhal, conjunctivis, with enormous chemosis, and infiltration of the cornea with lymph, and a central ulceration occurred, then resolution took place, and finally central albugo was left. After simple acu-puncture, in order to accustom the eye in some measure, the electro puncture was used. After four sittings, of some minutes each, at least two-thirds of the exuded matter were removed, but the pain was so severe at each application that the patient would not continue the remedy.

